# Bis{(*Z*)-[(*E*)-2-(pyridin-2-yl­methyl­idene)hydrazin-1-yl­idene][(pyridin-2-yl)methyl­sulfan­yl]methane­thiol­ato}nickel(II)

**DOI:** 10.1107/S1600536813013032

**Published:** 2013-05-18

**Authors:** Teng-Jin Khoo, Mohammed Khaled bin Break, M. Ibrahim M. Tahir, Karen A. Krouse, Andrew R. Cowley, David J. Watkin

**Affiliations:** a School of Pharmacy, University of Nottingham Malaysia Campus, Selangor, Malaysia; b School of Chemical Sciences and Food Technology, Universiti Kebangsaan Malaysia, 43600 Bangi, Malaysia; c Department of Chemistry, Faculty of Science, Universiti Putra Malaysia, Malaysia; d Chemical Crystallography, Chemistry Research Laboratory, 12 Mansfield Road, Oxford OX1 3TA, England

## Abstract

The title compound, [Ni(C_13_H_11_N_4_S_2_)_2_], was obtained by the reaction of *S*-2-picolyldi­thio­carbazate and pyridine-2-carbaldehyde with nickel(II) acetate. The Ni^II^ atom is located on a twofold rotation axis and is bonded to four N atoms at distances of 2.037 (8) and 2.109 (9) Å, and to two S atoms at a distance of 2.406 (3) Å, leading to a distorted octa­hedral coordination. The angle between the mean planes of the coordinating moieties of the two symmetry-related tridentate ligands is 83.3 (2)°. In the crystal, complex mol­ecules are linked by weak C—H⋯S hydrogen bonds, π–π inter­actions between the pyridine rings [centroid–centroid distance = 3.775 (9) Å] and C—H⋯π inter­actions. The hydrogen-bonding inter­actions lead to the formation of layers parallel to (010); π–π inter­actions link these layers into a three-dimensional network.

## Related literature

For biological applications of Schiff base ligands and complexes derived from di­thio­carbaza­tes, see: Hossain *et al.* (1996 ▶); Tarafder *et al.* (2002 ▶); Crouse *et al.* (2004 ▶). For a related structure, see: Omar *et al.* (2012 ▶).
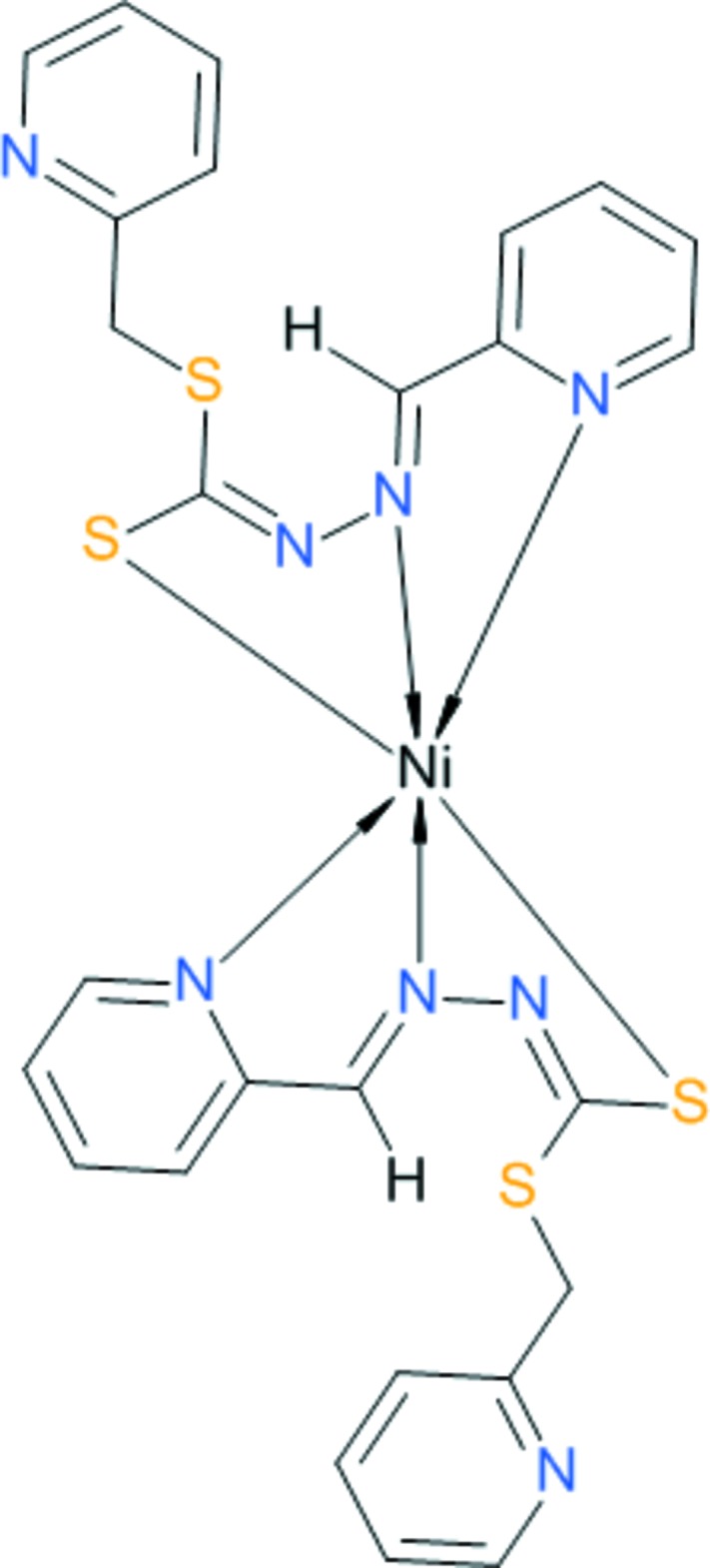


## Experimental

### 

#### Crystal data

[Ni(C_13_H_11_N_4_S_2_)_2_]
*M* *_r_* = 633.49
Monoclinic, 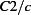

*a* = 26.0501 (4) Å
*b* = 8.0057 (1) Å
*c* = 13.0743 (2) Å
β = 103.8993 (9)°
*V* = 2646.80 (7) Å^3^
*Z* = 4
Mo *K*α radiation
μ = 1.08 mm^−1^
*T* = 150 K
0.04 × 0.03 × 0.02 mm

#### Data collection

Nonius Kappa CCD diffractometer
Absorption correction: multi-scan (*DENZO*/*SCALEPACK*; Otwinowski & Minor, 1997 ▶) *T*_min_ = 0.97, *T*_max_ = 0.98
5912 measured reflections
3030 independent reflections
2066 reflections with *I* > 3σ(*I*)
*R*_int_ = 0.020

#### Refinement

*R*[*F* ^2^ > 2σ(*F* ^2^)] = 0.045
*wR*(*F* ^2^) = 0.121
*S* = 1.08
2066 reflections
177 parameters
H-atom parameters constrained
Δρ_max_ = 0.81 e Å^−3^
Δρ_min_ = −0.74 e Å^−3^

### 

Data collection: *COLLECT* (Nonius, 2001 ▶); cell refinement: *DENZO*/*SCALEPACK* (Otwinowski & Minor, 1997 ▶); data reduction: *DENZO*/*SCALEPACK*; program(s) used to solve structure: *SIR92* (Altomare *et al.*, 1994 ▶); program(s) used to refine structure: *CRYSTALS* (Betteridge *et al.*, 2003 ▶); molecular graphics: *Mercury* (Macrae *et al.*, 2006 ▶); software used to prepare material for publication: *publCIF* (Westrip, 2010 ▶).

## Supplementary Material

Crystal structure: contains datablock(s) I, global. DOI: 10.1107/S1600536813013032/wm2742sup1.cif

Structure factors: contains datablock(s) I. DOI: 10.1107/S1600536813013032/wm2742Isup2.hkl

Supplementary material file. DOI: 10.1107/S1600536813013032/wm2742Isup3.mol

Additional supplementary materials:  crystallographic information; 3D view; checkCIF report

## Figures and Tables

**Table 1:** Hydrogen-bond geometry (Å, °) *Cg* is the centroid of the pyridine ring (C9–C13/N4).

*D*—H⋯*A*	*D*—H	H⋯*A*	*D*⋯*A*	*D*—H⋯*A*
C8—H7⋯S2^i^	0.94	2.72	3.644 (9)	166
C4—H4⋯S2^i^	0.95	2.92	3.862 (11)	175
C6—H6⋯*Cg* ^ii^	0.98	2.98	3.750 (12)	136

Symmetry codes: (i) 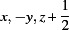; (ii) 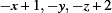.

**Fig. 1.**
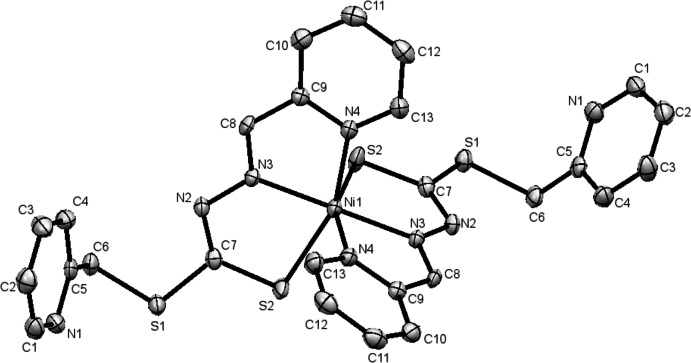
The molecular structure of the title compound showing 50% probability displacement ellipsoids in addition to the atomic numbering scheme. Hydrogen atoms were omitted for clarity. The second ligand is related to the first by symmetry code x, -y, z + 1/2.

**Fig. 2.**
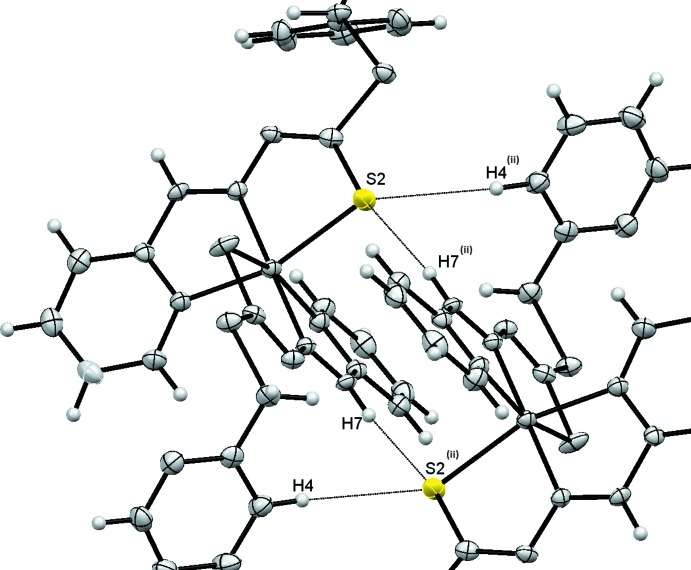
The molecules in the structure are stabilized by intermolecular C—H···S hydrogen bonding interactions. Probability function as in Fig. 1. [Symmetry code: (ii) x, -y, z + 1/2.]

**Fig. 3.**
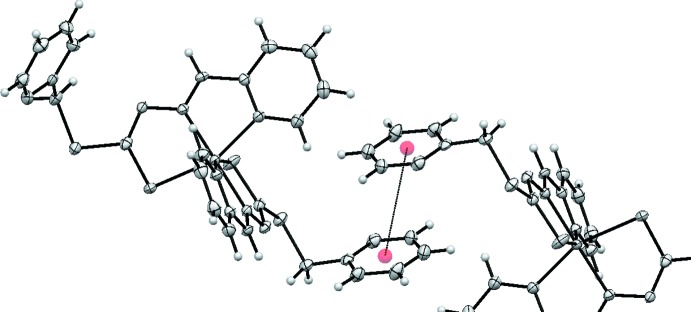
The molecules in the structure are also linked by π···π interactions between pairs of pyridine rings with a centroid···centroid distance of 3.775 (9) Å. Probability function as in Fig. 1. [Symmetry code: 3/2 - x, 1/2 - y, 2 - z.]

**Fig. 4.**
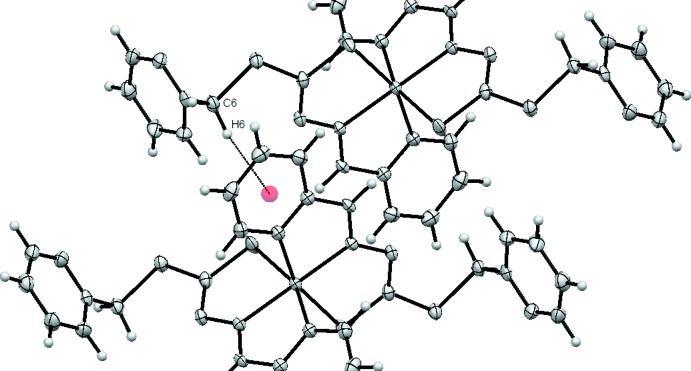
Diagram showing the C—H···π interaction between the molecules in the structure. Probability function as in Fig. 1. [Symmetry code: 1 - x, - y, 2 - z.]

## supplementary crystallographic information

### Comment

In the last few decades an increasing interest in the potential benefits of dithiocarbazates has arisen which has led to the synthesis of several Schiff base ligands and complexes that can be derived from dithiocarbazates (Tarafder *et al.*, 2002; Hossain *et al.*, 1996). S-2-picolyl dithiocarbazate (S2PDTC) is one type of a dithiocarbazate compound that has been synthesized recently, and its Schiff bases and complexes have proven to possess antimicrobial and anticancer activities (Crouse *et al.*, 2004). Due to these potential medicinal properties of S2PDTC-derived Schiff bases and complexes, the title compound was synthesized and structurally analyzed.

The Ni^II^ atom is situated on a twofold rotation axis and is bonded to four nitrogen atoms [Ni—N3 = 2.037 (8) Å; Ni—N4 = 2.109 (9) Å] and two sulfur atoms [Ni—S2 = 2.406 (3) Å] in a distorted octahedral coordination environment as exemplified by the angle N4—Ni1—S2 = 158.4 (2)° (Fig. 1). The bond length of C7—S2 is 1.723 (10) Å, similar to that of C7—S1 of 1.746 (10) Å, which indicates that the ligand bonds to the Ni^II^ ion in its thiol tautomer *via* the deprotonated S atoms.

The angle between the mean plane defined by (Ni1—S2—C7—N2—N3—N4—C8) and that of the symmetry-related ligand is 83.3 (2)° which shows that the two ligands are nearly orthogonal to each other. The Ni—N bond lengths of the title complex are very similar to that of a previously reported related structure (Omar *et al.*, 2012) [2.013 (2) Å for Ni—N, 2.179 (2) Å for Ni—N where this N atom belongs to the pyridine ring; 2.426 (7) Å for Ni—S] which might indicate that the values of such bond lengths are typical of nickel(II) complexes derived from dithiocarbazates. The pyridine ring (C1—C2—C3—C4—C5—N1) is nearly perpendicular to the rest of the molecule with a torsion angle of C5—C6—S1—C7 = 84.0 (8)°.

The molecules in the crystal are stabilized by weak intermolecular C—H···S hydrogen bonding interactions (Table 1; Fig. 2). Moreover, the pyridine rings (C1—C2—C3—C4—C5—N1) at (*x*, *y, z*) and (3/2 - *x*, 1/2 - *y*, 2 - *z*) are stacked parallel to each other and form π···π interactions (Fig. 3) with a centroid-centroid separation of 3.775 (9) Å and a shift distance of 1.878 (17) Å while the distance between the planes of the rings is 3.275 (12) Å. There are also C—H···π interactions (Table 1; Fig. 4).

### Experimental

The nickel complex was synthesized according to a modified procedure reported by Crouse *et al.* (2004): 0.02 mole of S-picolyl dithiocarbazate were added to a beaker containing 40 ml of absolute ethanol followed by heating the mixture on a heating plate with constant stirring in order to ensure complete dissolving. Similarly, 0.02 mole of pyridine-2-carbaldehyde were dissolved in a separate beaker containing 40 ml of absolute ethanol followed by heating and stirring of the mixture. The reactants were later mixed and 2–4 drops of concentrated H_2_SO_4_ were added to the mixture followed by heating of the mixture for 5 minutes. The mixture was cooled to 273 K in an ice-bath until the precipitation of the Schiff base ligand was achieved, and this was followed by filtration of the precipitated Schiff base ligand *via* suction filtration, washing it with cold ethanol and drying over silica gel.

0.0076 mole of the synthesized Schiff base ligand were dissolved in 50 ml of absolute ethanol followed by the addition of an equimolar amount of KOH and the mixture was heated over a heating plate and stirred until the compounds had been completely dissolved. The solution was then treated with a stoichiometric amount of nickel(II) acetate (0.0038 moles) dissolved in 50 ml of absolute ethanol followed by heating for 5 minutes and then kept in an ice-salt bath. Finally, the obtained product was isolated *via* suction filtration, washed with ethanol and dried over silica gel.

### Refinement

The H atoms were all located in a difference map, but those attached to carbon atoms were repositioned geometrically. The H atoms were initially refined with soft restraints on the bond lengths and angles to regularize their geometry (C—H in the range 0.93–98 Å) and isotropic temperature factors (*U*_iso_(H) in the range 1.2–1.5 times *U*_eq_ of the parent atom), after which the positions were refined with riding constraints.

### Crystal data

**Table:**

[Ni(C_13_H_11_N_4_S_2_)_2_]	*F*(000) = 1304
*M**_r_* = 633.49	*D*_x_ = 1.590 Mg m^−^^3^
Monoclinic, *C*2/*c*	Mo *K*α radiation, λ = 0.71073 Å
Hall symbol: -C 2yc	Cell parameters from 3175 reflections
*a* = 26.0501 (4) Å	θ = 1–27°
*b* = 8.0057 (1) Å	µ = 1.08 mm^−^^1^
*c* = 13.0743 (2) Å	*T* = 150 K
β = 103.8993 (9)°	Plate, dark green
*V* = 2646.80 (7) Å^3^	0.04 × 0.03 × 0.02 mm
*Z* = 4	

### Data collection

**Table:**

Nonius Kappa CCD diffractometer	2066 reflections with *I* > 3σ(*I*)
Graphite monochromator	*R*_int_ = 0.020
ω scans	θ_max_ = 27.5°, θ_min_ = 2.7°
Absorption correction: multi-scan (*DENZO*/*SCALEPACK*; Otwinowski & Minor, 1997)	*h* = −33→33
*T*_min_ = 0.97, *T*_max_ = 0.98	*k* = −10→10
5912 measured reflections	*l* = −16→16
3030 independent reflections	

### Refinement

**Table:**

Refinement on *F*	Primary atom site location: structure-invariant direct methods
Least-squares matrix: full	Hydrogen site location: inferred from neighbouring sites
*R*[*F*^2^ > 2σ(*F*^2^)] = 0.045	H-atom parameters constrained
*wR*(*F*^2^) = 0.121	Method = Quasi-Unit *w*eights W = 1.0 or 1./2*F*
*S* = 1.08	(Δ/σ)_max_ = 0.000256
2066 reflections	Δρ_max_ = 0.81 e Å^−^^3^
177 parameters	Δρ_min_ = −0.74 e Å^−^^3^
0 restraints	

### Fractional atomic coordinates and isotropic or equivalent isotropic displacement parameters (Å^2^)

**Table:**

	*x*	*y*	*z*	*U*_iso_*/*U*_eq_	
H1	0.8234	0.1314	1.0674	0.0390*	
H2	0.8039	0.3461	1.1740	0.0419*	
H3	0.7243	0.3331	1.2252	0.0430*	
H4	0.6648	0.1115	1.1674	0.0380*	
H5	0.6999	−0.2613	1.0830	0.0320*	
H6	0.6483	−0.1561	1.0864	0.0320*	
H7	0.5288	0.1849	1.0417	0.0250*	
H8	0.4519	0.3855	1.0530	0.0351*	
H9	0.3830	0.5632	0.9638	0.0430*	
H10	0.3638	0.5694	0.7768	0.0412*	
H11	0.4110	0.4002	0.6869	0.0369*	
C1	0.7910 (4)	0.1272 (16)	1.0890 (8)	0.0279	
C2	0.7800 (5)	0.2562 (16)	1.1526 (9)	0.0301	
C3	0.7328 (5)	0.2491 (16)	1.1824 (9)	0.0304	
C4	0.6977 (4)	0.1170 (16)	1.1487 (8)	0.0266	
C5	0.7126 (4)	−0.0081 (14)	1.0863 (7)	0.0204	
C6	0.6789 (4)	−0.1619 (14)	1.0548 (8)	0.0229	
C7	0.5986 (4)	−0.0675 (13)	0.8762 (8)	0.0197	
C8	0.5162 (4)	0.1936 (13)	0.9682 (7)	0.0177	
C9	0.4708 (4)	0.2967 (13)	0.9196 (7)	0.0178	
C10	0.4431 (4)	0.3919 (15)	0.9783 (8)	0.0243	
C11	0.4023 (4)	0.4963 (15)	0.9253 (9)	0.0281	
C12	0.3908 (4)	0.4991 (15)	0.8152 (9)	0.0284	
C13	0.4192 (4)	0.3980 (16)	0.7618 (8)	0.0253	
S1	0.65548 (10)	−0.1902 (4)	0.9126 (2)	0.0223	
S2	0.56873 (11)	−0.0826 (4)	0.7438 (2)	0.0273	
N1	0.7591 (3)	−0.0039 (12)	1.0560 (7)	0.0250	
N2	0.5828 (3)	0.0209 (11)	0.9494 (6)	0.0185	
N3	0.5379 (3)	0.1138 (11)	0.9056 (6)	0.0168	
N4	0.4582 (3)	0.2990 (11)	0.8126 (6)	0.0196	
Ni1	0.5000	0.1151 (3)	0.7500	0.0172	

### Atomic displacement parameters (Å^2^)

**Table:**

	*U*^11^	*U*^22^	*U*^33^	*U*^12^	*U*^13^	*U*^23^
C1	0.019 (5)	0.037 (7)	0.026 (5)	−0.004 (5)	0.002 (4)	0.004 (5)
C2	0.027 (6)	0.032 (6)	0.028 (6)	−0.007 (5)	0.000 (5)	−0.001 (5)
C3	0.025 (6)	0.034 (6)	0.029 (6)	0.004 (5)	0.002 (5)	−0.012 (5)
C4	0.022 (5)	0.034 (6)	0.023 (5)	0.002 (5)	0.005 (4)	−0.006 (5)
C5	0.019 (5)	0.024 (5)	0.015 (4)	0.004 (4)	−0.002 (4)	0.005 (4)
C6	0.022 (5)	0.026 (6)	0.018 (5)	0.001 (4)	−0.001 (4)	0.004 (4)
C7	0.019 (5)	0.017 (5)	0.020 (5)	−0.002 (4)	0.000 (4)	0.000 (4)
C8	0.022 (5)	0.020 (5)	0.010 (4)	−0.002 (4)	0.001 (4)	−0.002 (4)
C9	0.019 (5)	0.016 (5)	0.018 (5)	0.000 (4)	0.004 (4)	0.000 (4)
C10	0.026 (5)	0.025 (6)	0.023 (5)	−0.005 (5)	0.007 (4)	−0.002 (5)
C11	0.025 (5)	0.026 (6)	0.035 (6)	−0.002 (5)	0.012 (5)	−0.005 (5)
C12	0.021 (5)	0.025 (6)	0.039 (6)	0.003 (5)	0.005 (5)	0.004 (5)
C13	0.022 (5)	0.031 (6)	0.021 (5)	0.004 (5)	0.002 (4)	0.002 (5)
S1	0.0207 (12)	0.0238 (14)	0.0197 (12)	0.0040 (11)	−0.0003 (9)	−0.0014 (11)
S2	0.0276 (14)	0.0357 (17)	0.0145 (11)	0.0112 (12)	−0.0026 (10)	−0.0066 (11)
N1	0.019 (4)	0.032 (5)	0.023 (4)	0.005 (4)	0.003 (3)	−0.001 (4)
N2	0.018 (4)	0.019 (4)	0.016 (4)	0.002 (4)	−0.001 (3)	0.001 (3)
N3	0.017 (4)	0.018 (4)	0.014 (4)	−0.001 (4)	0.002 (3)	−0.002 (3)
N4	0.019 (4)	0.023 (5)	0.016 (4)	0.001 (4)	0.003 (3)	0.001 (4)
Ni1	0.0172 (9)	0.0210 (10)	0.0120 (8)	0.0000	0.0007 (6)	0.0000

### Geometric parameters (Å, º)

**Table:**

H1—C1	0.952	C5—N1	1.361 (13)
H2—C2	0.948	C6—S1	1.826 (10)
H3—C3	0.936	C7—S1	1.746 (10)
H4—C4	0.949	C7—S2	1.723 (10)
H5—C6	0.986	C7—N2	1.333 (13)
H6—C6	0.982	C8—C9	1.458 (14)
H7—C8	0.940	C8—N3	1.272 (13)
H8—C10	0.949	C9—C10	1.398 (14)
H9—C11	0.956	C9—N4	1.358 (12)
H10—C12	0.944	C10—C11	1.399 (16)
H11—C13	0.951	C11—C12	1.398 (16)
C1—C2	1.397 (17)	C12—C13	1.392 (16)
C1—N1	1.345 (15)	C13—N4	1.335 (13)
C2—C3	1.377 (16)	S2—Ni1	2.406 (3)
C3—C4	1.398 (17)	N2—N3	1.388 (11)
C4—C5	1.404 (15)	N3—Ni1	2.037 (8)
C5—C6	1.512 (15)	N4—Ni1	2.109 (9)
			
H1—C1—C2	118.2	H9—C11—C12	121.5
H1—C1—N1	117.1	C10—C11—C12	118.0 (10)
C2—C1—N1	124.6 (10)	C11—C12—H10	120.4
H2—C2—C1	121.6	C11—C12—C13	119.9 (10)
H2—C2—C3	120.9	H10—C12—C13	119.7
C1—C2—C3	117.5 (11)	H11—C13—C12	119.4
H3—C3—C2	119.8	H11—C13—N4	118.5
H3—C3—C4	120.0	C12—C13—N4	122.0 (10)
C2—C3—C4	120.2 (11)	C6—S1—C7	105.2 (5)
H4—C4—C3	121.2	C7—S2—Ni1	94.6 (4)
H4—C4—C5	120.7	C5—N1—C1	116.8 (9)
C3—C4—C5	118.1 (10)	C7—N2—N3	111.3 (8)
C4—C5—C6	121.0 (9)	N2—N3—C8	117.6 (8)
C4—C5—N1	122.8 (10)	N2—N3—Ni1	124.9 (6)
C6—C5—N1	116.1 (9)	C8—N3—Ni1	117.2 (7)
C5—C6—H5	108.8	C9—N4—C13	119.1 (9)
C5—C6—H6	108.9	C9—N4—Ni1	111.8 (7)
H5—C6—H6	108.4	C13—N4—Ni1	128.7 (7)
C5—C6—S1	114.1 (7)	N4—Ni1—N4^i^	91.5 (5)
H5—C6—S1	107.4	N4—Ni1—S2	158.4 (2)
H6—C6—S1	109.1	N4^i^—Ni1—S2	89.4 (2)
S1—C7—S2	112.5 (6)	N4—Ni1—S2^i^	89.4 (2)
S1—C7—N2	119.4 (7)	N4^i^—Ni1—S2^i^	158.4 (2)
S2—C7—N2	128.0 (8)	S2—Ni1—S2^i^	97.72 (17)
H7—C8—C9	122.4	N4—Ni1—N3	77.7 (3)
H7—C8—N3	121.3	N4^i^—Ni1—N3	102.7 (3)
C9—C8—N3	116.3 (8)	S2—Ni1—N3	81.0 (2)
C8—C9—C10	122.7 (9)	S2^i^—Ni1—N3	98.6 (2)
C8—C9—N4	115.2 (9)	N4—Ni1—N3^i^	102.7 (3)
C10—C9—N4	122.1 (9)	N4^i^—Ni1—N3^i^	77.7 (3)
H8—C10—C9	120.4	S2—Ni1—N3^i^	98.6 (2)
H8—C10—C11	120.6	S2^i^—Ni1—N3^i^	81.0 (2)
C9—C10—C11	119.0 (10)	N3—Ni1—N3^i^	179.4 (5)
H9—C11—C10	120.5		

Symmetry code: (i) −*x*+1, *y*, −*z*+3/2.

### Hydrogen-bond geometry (Å, º)

Cg is the centroid of the pyridine ring (C9–C13/N4).

**Table:**

*D*—H···*A*	*D*—H	H···*A*	*D*···*A*	*D*—H···*A*
C8—H7···S2^ii^	0.94	2.72	3.644 (9)	166
C4—H4···S2^ii^	0.95	2.92	3.862 (11)	175
C6—H6···*Cg*^iii^	0.98	2.98	3.750 (12)	136

Symmetry codes: (ii) *x*, −*y*, *z*+1/2; (iii) −*x*+1, −*y*, −*z*+2.

## References

[bb1] Altomare, A., Cascarano, G., Giacovazzo, C., Guagliardi, A., Burla, M. C., Polidori, G. & Camalli, M. (1994). *J. Appl. Cryst.* **27**, 435.

[bb2] Betteridge, P. W., Carruthers, J. R., Cooper, R. I., Prout, K. & Watkin, D. J. (2003). *J. Appl. Cryst.* **36**, 1487.

[bb3] Crouse, K. A., Chew, -B., Tarafder, M., Kasbollah, A., Ali, M., Yamin, M. & Fun, H. K. (2004). *Polyhedron*, **23**, 161–168.

[bb4] Hossain, E., Alam, M., Ali, M., Nazimuddin, M., Smith, E. & Hynes, C. (1996). *Polyhedron*, **15**, 973–980.

[bb5] Macrae, C. F., Edgington, P. R., McCabe, P., Pidcock, E., Shields, G. P., Taylor, R., Towler, M. & van de Streek, J. (2006). *J. Appl. Cryst.* **39**, 453–457.

[bb6] Nonius (2001). *COLLECT* Nonius BV, Delft, The Netherlands.

[bb7] Omar, S. A., Ravoof, T. B. S. A., Mohamed Tahir, M. I. & Crouse, K. A. (2012). *Acta Cryst.* E**68**, m316–m317. 10.1107/S1600536812006952 PMC3297266 22412456

[bb8] Otwinowski, Z. & Minor, W. (1997). *Methods in Enzymology*, Vol. 276, *Macromolecular Crystallography*, Part A, edited by C. W. Carter Jr & R. M. Sweet, pp. 307–326. New York: Academic Press.

[bb9] Tarafder, M., Khoo, T. J., Crouse, K. A., Ali, M., Yamin, B. & Fun, H.-K. (2002). *Polyhedron*, **21**, 2691–2698.

[bb10] Westrip, S. P. (2010). *J. Appl. Cryst.* **43**, 920–925.

